# Continuous inhibition of epidermal growth factor receptor phosphorylation by erlotinib enhances antitumor activity of chemotherapy in erlotinib-resistant tumor xenografts

**DOI:** 10.3892/or.2011.1614

**Published:** 2011-12-30

**Authors:** TOSHIKI IWAI, YOICHIRO MORIYA, MASATOSHI SHIRANE, KAORI FUJIMOTO-OUCHI, KAZUSHIGE MORI

**Affiliations:** Product Research Department, Chugai Pharmaceutical Co., Ltd., 200 Kajiwara, Kamakura 247-8530, Japan

**Keywords:** erlotinib, docetaxel, irinotecan, resistance, epidermal growth factor receptor, non-small cell lung cancer, pancreatic cancer, progressive disease

## Abstract

Erlotinib, an epidermal growth factor receptor tyrosine kinase inhibitor, has been shown to have benefits for non-small cell lung cancer and pancreatic cancer patients; however, almost all patients develop progressive disease during the therapy. On the other hand, it has been reported that a tumor continues to express epidermal growth factor receptor even after developing progressive disease. To demonstrate the clinical relevance of erlotinib treatment after progressive disease, we investigated whether continuous administration of erlotinib in combination with chemotherapy has a useful effect on progressive disease development during erlotinib treatment. For this purpose, we examined the antitumor effect of a combination therapy of a chemotherapeutic agent with erlotinib using two types of erlotinib-resistant tumor xenograft models: a non-small cell lung cancer model, in which EBC-1, H1975 and HCC827TR3 tumors were implanted, and an HPAC pancreatic cancer cell xenograft which generates erlotinib-resistant tumors *in vivo*. As a result, the combination therapy showed a significantly higher antitumor activity compared with chemomonotherapy in all xenograft models except the H1975 xenografts. Furthermore, erlotinib alone suppressed the phosphorylation of epidermal growth factor receptor in HPAC tumors and the two non-small cell lung cancer cell lines other than H1975. Therefore, combination therapy which uses erlotinib can be considered effective if epidermal growth factor receptor phosphorylation is inhibited by erlotinib, even in erlotinib-resistant tumor xenograft models. Our results suggest that the continuous inhibition of epidermal growth factor receptor phosphorylation by erlotinib after progressive disease enhances the antitumor activity of chemotherapy.

## Introduction

The epidermal growth factor receptor (EGFR) is a transmembrane glycoprotein with an extracellular EGF-binding domain and an intracellular domain possessing intrinsic tyrosine kinase activity (1,2). Ligand binding activates the receptor's tyrosine kinase, initiating cascades of intracellular signaling such as those via the Ras protein (3). High levels of EGFR expression have been reported in a wide range of human malignancies (4–6) and enhanced expression of EGFR has previously been shown in non-small cell lung cancer (NSCLC) (7).

Since it was reported that EGFR overexpression is a factor of poor prognosis (8,9), treatments targeting EGFR would be expected to show survival benefits. Erlotinib (Tarceva^®^) is an oral, small molecule tyrosine kinase inhibitor that reversibly binds to the intracellular domain of EGFR. This blocks autophosphorylation of EGFR with subsequent inhibition of the downstream signaling pathways which promote cell proliferation. Erlotinib is used for metastatic NSCLC and pancreatic cancer in many countries. Clinical results have demonstrated that erlotinib monotherapy or combination therapy with gemcitabine showed a survival benefit for NSCLC or pancreatic cancer, respectively (10,11). However, most of these patients developed progressive disease (PD) during such therapies and it is usually considered best to switch to chemomonotherapy after developing PD. It is reported that the major mechanisms of erlotinib resistance are gatekeeper mutation (T790M) of EGFR and *c-Met* amplification (12,13) in tumor cells. On the other hand, it is reported that the tumor cells express active EGFR even after acquiring resistance to erlotinib (13,14). Considering that EGFR overexpression is a factor of poor prognosis, discontinuing erlotinib treatment after PD has developed may be an inappropriate option and combining erlotinib with the next stage of chemotherapy may be an appropriate therapy. We have previously reported that the combination of docetaxel with erlotinib showed a synergistic effect in NSCLC cell lines *in vivo* irrespective of EGFR or K-RAS mutation status (15).

Therefore, we investigated the antitumor effect of combination therapies of erlotinib with various chemotherapeutic agents docetaxel, irinotecan and gemcitabine, using erlotinib-resistant tumor cell xenografts as well as an *in vivo* erlotinib PD xenograft model, to show the clinical relevance of continuing erlotinib treatment after development of PD.

## Materials and methods

### Chemicals

Erlotinib was provided by F. Hoffman-La Roche (Basel, Switzerland) as a fine powder and was dissolved in distilled water containing 6% (w/v) Captisol (CyDex Pharmaceuticals, KS, USA) and diluted with saline for *in vivo* experiments. Erlotinib was dissolved in DMSO for *in vitro* experiments. Docetaxel was synthesized by Kanto Chemical Co., Inc. (Tokyo, Japan) as a fine powder and was dissolved in saline containing 2.5% (v/v) polysorbate 80 (Sigma-Aldrich Co., USA) and 2.5% (v/v) ethanol for *in vivo* experiments. Irinotecan was purchased from Daiichi Sankyo Pharmaceutical Co., Ltd. (Tokyo, Japan) as an aqueous solution and diluted with saline.

### Animals

Male 5-week-old BALB-nu/nu mice (CAnN.Cg-Foxn1<nu>/CrlCrlj nu/nu) were obtained from Charles River Japan (Kanagawa, Japan). All animals were allowed to acclimatize and recover from shipping-related stress for 1 week prior to the study. The health of the mice was monitored by daily observation. Chlorinated water and irradiated food were provided *ad libitum*, and the animals were kept in a controlled light-dark cycle (12 h-12 h). The protocol was reviewed by the Institutional Animal Care and Use Committee of Chugai Pharmaceutical Co., Ltd., and all mouse experiments were performed in accordance with the Guidelines for the Accommodation and Care of Laboratory Animals promulgated in Chugai Pharmaceutical Co., Ltd.

### Tumor cells

Human non-small cell lung cancer (NSCLC) cell lines, HCC827 (exon 19 deletion EGFR) and H1975 (T790M mutation in EGFR), and human pancreatic cancer cell line, HPAC (wild-type EGFR), were obtained from the American Type Culture Collection. Human NSCLC cell line, EBC-1 (*c-Met*-amplification) was obtained from the RIKEN BRC (Ibaraki, Japan). Erlotinib-resistant cell line HCC827TR3 was established in-house by exposing HCC827 cells to increasing concentrations of erlotinib *in vitro*. The HCC827, HCC827TR3 and H1975 cells were maintained at 37°C under 5% CO_2_ in RPMI-1640 medium (Sigma-Aldrich Co.) containing 10% FBS, 10 mM HEPES, 1 mM sodium pyruvate, and 4.5 g/l glucose. The HPAC cell line was maintained in DMEM: Ham's F12 combined medium (1:1) (Invitrogen, USA) containing 5% FBS, 2 μg/ml insulin, 5 μg/ml transferrin, 40 ng/ml hydrocortisone, and 10 ng/ml EGF. The EBC-1 was maintained in EMEM (Sigma-Aldrich Co.) containing 10% FBS.

### Evaluation of antitumor activity

#### Study 1

HCC827TR3, EBC-1, H1975 xenograft models and treatment. A suspension of tumor cells (5×10^6^ cells/mouse) was inoculated subcutaneously into the right flank of mice. Tumors were allowed to reach 0.1–0.3 cm^3^ in size, mice were randomly allocated to the control group, erlotinib group, chemotherapy group and combination of erlotinib with chemotherapy group and these were treated with vehicle of erlotinib and vehicle of chemotherapy, erlotinib and vehicle of chemotherapy, vehicle of erlotinib and chemotherapy, or erlotinib and chemotherapy, respectively. Erlotinib was administered orally (p.o.) once a day from Day 2. Docetaxel was administered intravenously (i.v.) once in 3 weeks (Day 1). Irinotecan was administered intravenously (i.v.) once in 2 weeks (Day 1). To evaluate the antitumor effect and tolerability, tumor volume and body weight were measured twice a week. The tumor volume (V) was estimated from the equation V = ab^2^/2, where a and b were tumor length and width, respectively.

#### Study 2

Establishment of *in vivo* erlotinib PD model and treatment. To establish an *in vivo* erlotinib PD model, a suspension of HPAC cells (5×10^6^ cells/mouse) was inoculated subcutaneously into the right flank of the mice. Tumors were allowed to reach 0.1–0.3 cm^3^ in size, mice were randomly allocated to control and erlotinib groups. Erlotinib was administered orally (p.o.) once a day starting from Day 1 to Day 18.

After establishment of PD during erlotinib treatment was confirmed, mice were re-randomized and allocated to the control group, erlotinib group, gemcitabine group, and combination of gemcitabine with erlotinib group and these were treated with vehicle of erlotinib and vehicle of gemcitabine, erlotinib and vehicle of gemcitabine, vehicle of erlotinib and gemcitabine, or erlotinib and gemcitabine, respectively. Erlotinib was administered orally (p.o.) on Days 21–25, 28–32, 35–40. Gemcitabine was administered i.v. once a week (on Days 20, 27 and 34). To evaluate the antitumor effect and tolerability, tumor volume and body weight were measured twice a week. The tumor volume (V) was estimated from the equation V = ab^2^/2, where a and b were tumor length and width, respectively.

#### Western blotting

Cells (HCC827, HCC827TR3, EBC-1 and H1975) were seeded into 6-well plates at a concentration of 5×10^5^ cells per well and were preincubated overnight. Then, erlotinib was added and incubation continued for 2 h. Cells were stimulated with 100 ng/ml of EGF (Invitrogen) for the last 15 min of the incubation. HPAC tumor tissues of the *in vivo* PD model were pulverized in liquid nitrogen. Cellular total protein was prepared from cell lysates and the pulverized frozen tumors. Proteins (100 μg each of HPAC, EBC-1 and H1975; 5 μg each of HCC827 and HCC827TR3) were electrophoresed on SDS-PAGE with 7.5% gel and transferred onto PVDF membranes (GE Healthcare Japan, Tokyo, Japan). The membranes were blocked with a blocking buffer (Thermo Fisher Scientific, Kanagawa, Japan), immunoblotted with primary antibody against EGFR (Santa Cruz Biotechnology Inc., CA, USA), pY1068 pEGFR (Cell Signaling Technology Inc.) and GAPDH (Santa Cruz Biotechnology Inc.). The protein-antibody complex was detected by chemiluminescence (GE Healthcare Japan).

#### Cell proliferation assay

Cells were seeded at a density of 1000 or 3000 cells/well in 96-well plates and were preincubated overnight. The cells were then treated with erlotinib for 96 h. Cell proliferation was evaluated by Cell Counting Kit-8 (Dojindo, Kumamoto, Japan).

#### Statistical analysis

Statistical analysis to evaluate the antitumor activity was performed using the Mann-Whitney U test. For *in vitro* experiments, Student's t-test was used. Differences were considered to be significant at P≤0.05. Statistical analysis was carried out using the SAS preclinical package (SAS Institute, Inc., Tokyo, Japan).

## Results

### Erlotinib sensitivity, EGFR expression and effect of erlotinib on phosphorylation of EGFR and downstream signaling molecules in erlotinib-resistant NSCLC cells

**Figure 1:**
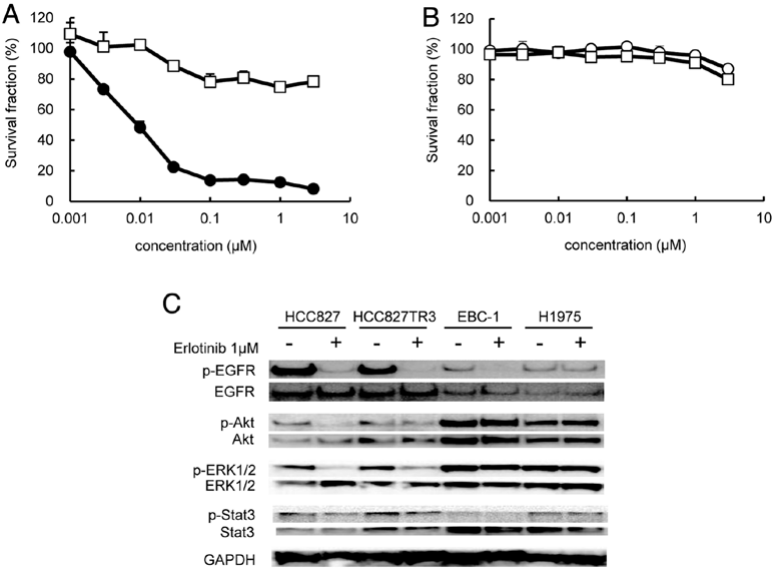
Erlotinib sensitivity, EGFR expression and effect of erlotinib on phosphorylation of EGFR and downstream signaling molecules in cancer cell lines *in vitro*. (A) Growth inhibition of erlotinib in parental HCC827 (●) and resistant HCC827TR3 (□). (B) Growth inhibition by erlotinib in EBC-1 (○) and H1975 (□). (C) Expression of EGFR and inhibition of phosphorylation in signal transduction molecules by erlotinib in NSCLC cell lines.

First, we examined the growth inhibition of tumor cells, namely HCC827, HCC827TR3, EBC-1 and H1975. HCC827TR3 was 1000 times more resistant to erlotinib than parental HCC827 (Fig. 1A) *in vitro*. We found that the mechanism of erlotinib resistance of HCC827TR3 was neither *c-Met* amplification nor T790M mutation in EGFR (data not shown). Almost no growth inhibition was observed in EBC-1 and H1975 cells up to 3 μmol/l of erlotinib (Fig. 1B). Next, we examined EGFR expression in the tumor cells and the effect of erlotinib on the phosphorylation of EGFR, as well as its major downstream signal molecules such as Akt, ERK, Stat3, by Western blotting. All of the cell lines expressed EGFR and phosphorylated EGFR (Fig. 1C). The EGFR phosphorylation was completely suppressed by erlotinib in HCC827, HCC827TR3 and EBC-1, although erlotinib did not inhibit the proliferation of HCC827TR3 and EBC-1. On the other hand, erlotinib did not suppress the phosphorylation of EGFR in H1975 cells (Fig. 1C). Erlotinib suppressed the phosphorylation of Akt and ERK in HCC827 cells. However, out of the three erlotinib-resistant cell lines, only a slight inhibition of ERK phosphorylation in HCC827TR3 was observed (Fig. 1C).

### Antitumor effect of combination therapy of chemotherapeutic agents with erlotinib in erlotinib-resistant tumor xenografts

Because EGFR phosphorylation was suppressed by erlotinib in the erlotinib-resistant cells (EBC-1, HCC827TR3), it may be of value to administer erlotinib concurrently with a chemotherapeutic agent when treating erlotinib-resistant tumors. Therefore, we next examined the antitumor activity of combination therapy of a chemotherapeutic agent with erlotinib against these erlotinib-resistant cell lines in xenografts.

**Figure 2:**
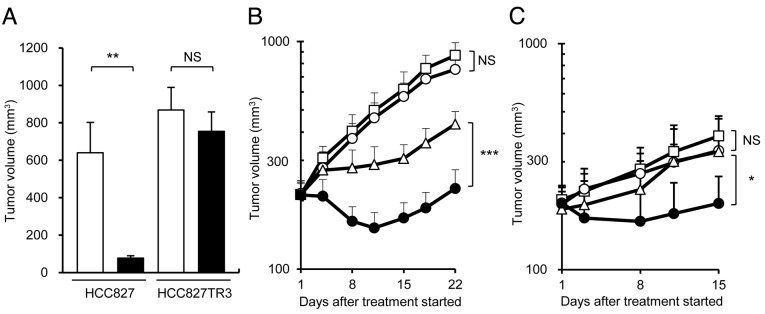
Antitumor effect in parental HCC827 and resistant HCC827TR3 xenograft models. (A) Erlotinib monotherapy at Day 22. □, control; ■, erlotinib 15 mg/kg (HCC827), 25 mg/kg (HCC827TR3), n=5/group. (B) Combination therapy of docetaxel with erlotinib in HCC827TR3 xenograft model. □, control; ○, erlotinib 25 mg/kg; ▵, docetaxel 20 mg/kg; ●, combination, n=7/group. (C) Combination therapy of irinotecan with erlotinib in HCC827TR3 xenograft model. □, control; ○, erlotinib 25 mg/kg; ▵, irinotecan 60 mg/kg; ●, combination, n=5/group. Statistically significant differences are shown. NS, not significant; ^*^P≤0.05, ^**^P≤0.01, ^***^P≤0.001 by Wilcoxon test.

**Figure 3:**
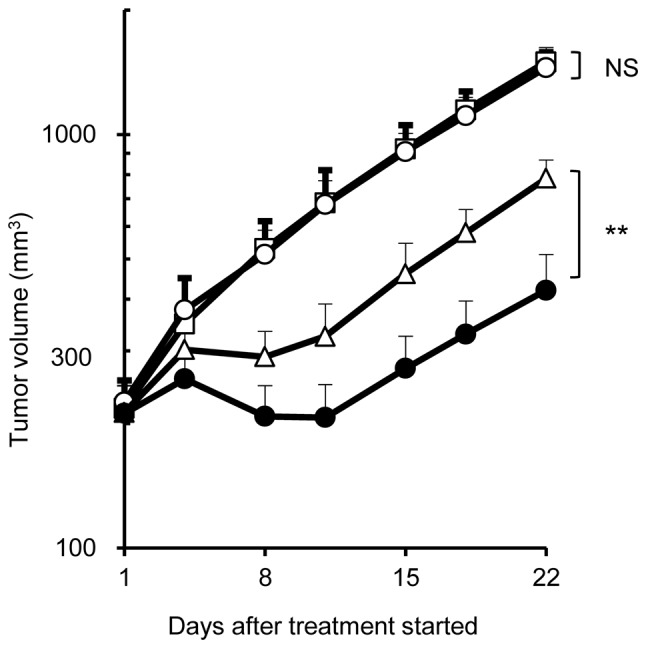
Antitumor effect of docetaxel in combination with erlotinib in EBC-1 xenograft model. □, control; ○, erlotinib 75 mg/kg; ▵, docetaxel 5 mg/kg; ●, combination, n=6/group. Statistically significant differences are shown. NS, not significant; ^**^P≤0.01 by Wilcoxon test.

**Figure 4:**
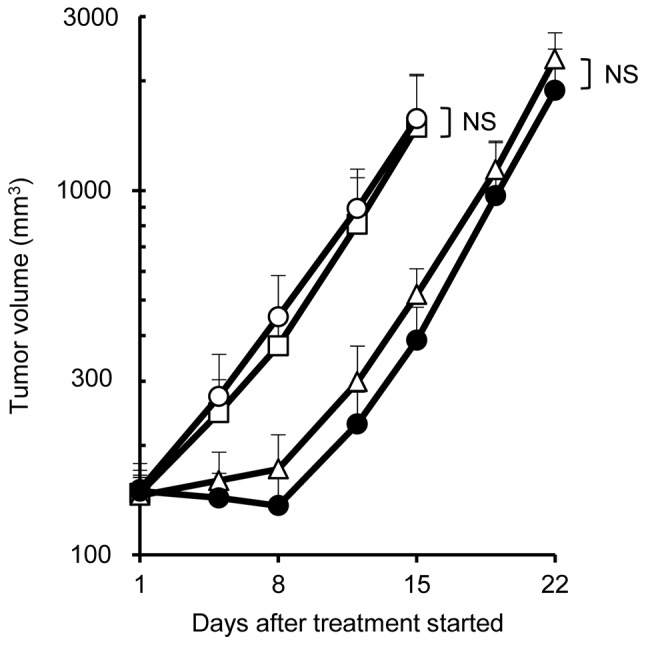
Antitumor effect of docetaxel in combination with erlotinib in H1975 xenograft model. □, control; ○, erlotinib 75 mg/kg; ▵, docetaxel 5 mg/kg; ●, combination, n=7/group. Statistically significant differences are shown. NS, not significant.

First, we examined the antitumor effect of docetaxel monotherapy and docetaxel + erlotinib therapy using the HCC827TR3 xenograft model. In this model, erlotinib monotherapy did not show any antitumor effect even at a dose of 25 mg/kg, which was higher than the effective dose for parental HCC827 xenograft model (Fig. 2A). However, docetaxel in combination with erlotinib showed a significantly higher antitumor activity compared with docetaxel monotherapy (Fig. 2B). A similar result was obtained in the combination therapy of irinotecan with erlotinib in the same xenograft model (Fig. 2C). In the EBC-1 xenograft model, similarly, significantly higher antitumor effect was obtained in the combination therapy of docetaxel (5 mg/kg) with erlotinib (75 mg/kg) compared to docetaxel monotherapy whereas erlotinib did not show any antitumor effect at the same dose (Fig. 3). Namely, the combination therapy of chemotherapeutic agent with erlotinib showed a significantly higher antitumor effect compared with chemomonotherapy while erlotinib monotherapy showed no effect in HCC827TR3 or EBC-1 xenografts. On the other hand, no significant effect was seen between docetaxel monotherapy (5 mg/kg) and combination of docetaxel (5 mg/kg) with erlotinib (75 mg/kg) in the H1975 xenograft model (Fig. 4).

### Establishment of in vivo erlotinib-resistant model and antitumor activity of gemcitabine in combination with erlotinib

**Figure 5:**
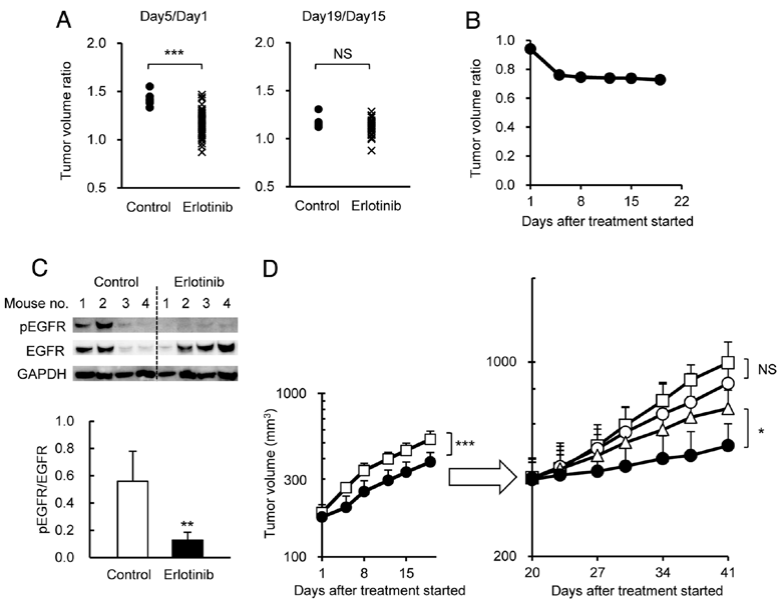
Establishment of an *in vivo* erlotinib-resistant model and antitumor activity of gemcitabine in combination with erlotinib. (A) Ratio between tumor volume and that of 4 days previously. Appearance of the progressive tumor during 19 days of treatment with erlotinib is shown. Mice were allocated to groups of 6 mice for control and 70 mice for erlotinib treatment. Erlotinib was administered p.o. qd for 19 days. ●, control; x, erlotinib 75 mg/kg. (B) Time course of the tumor volume ratio of erlotinib group to vehicle group. (C) Expression and phosphorylation of EGFR after acquiring resistance. Tumor tissues after acquiring resistance to erlotinib treatment were collected on Day 21. (D) Antitumor activity of gemcitabine in combination with erlotinib. On Day 20 of erlotinib treatment, mice in the erlotinib group were randomly allocated to 4 groups (n=6/group). □, control; ○, erlotinib 75 mg/kg; ▵, gemcitabine 25 mg/kg; ●, combination. Statistically significant differences are shown. NS, not significant. ^***^P≤0.001, ^*^P≤0.05 by Wilcoxon test.

To mimic the clinical PD phenomenon and examine the effect of combination therapy of docetaxel with erlotinib, we established an *in vivo* erlotinib-resistant model using EGFR-positive pancreatic cancer cell line HPAC. The HPAC cells were subcutaneously inoculated into BALB/c-nu/nu mice, and erlotinib (75 mg/kg) was administered p.o. once a day for 18 days. In this model, erlotinib significantly inhibited tumor growth up to 5 days after the start of administration (Fig. 5A). Subsequently, however, the tumor growth inhibition effect by erlotinib disappeared, even though erlotinib was continuously administered (Fig. 5A). Fig. 5B shows the constant tumor volume ratio of erlotinib group to vehicle group after around Day 8. On Day 20, the mice in the erlotinib group were randomly allocated to 4 groups, namely, vehicle group, erlotinib group, gemcitabine group, and gemcitabine + erlotinib group. Although EGFR protein remained positive and its phosphorylation had been substantially reduced by erlotinib by Day 21 (Fig. 5C), the erlotinib group did not show significant tumor growth inhibition compared with the vehicle group (Fig. 5D). This indicated that the HPAC tumors had become resistant to erlotinib.

Using this model, we examined the antitumor activity of combination therapy of gemcitabine (25 mg/kg) with erlotinib (75 mg/kg). The results indicated that the combination therapy showed a significant antitumor effect compared with gemcitabine monotherapy (Fig. 5D) even though erlotinib monotherapy showed no tumor inhibitory effect.

## Discussion

By using two types of tumor models, we were able to investigate the mechanism by which NSCLC and pancreatic cancer become resistant to erlotinib. Although EBC-1 and H1975 show amplification of c-Met and mutation of T790M, respectively, HCC827TR3, which was established in-house, has neither. In the HCC827TR3 cells, neither EGFR down-regulation nor reduction of EGFR phosphorylation was observed (Fig. 1C). The fact that EGFR phosphorylation was inhibited by erlotinib in HCC827TR3 cells but the PI3K pathway was not inhibited and the Ras-ERK/MAPK pathway only partially inhibited (Fig. 1C) indicates that the resistance mechanism may be the activation of these pathways by protein kinase(s) other than c-MET.

Erlotinib completely inhibited EGFR phosphorylation in EBC-1 and HCC827TR3 cells but not in H1975 cells (Fig. 1C). This coincides well with the previous reports (12,13,14) which state that, in cells with *c-Met* amplification, erlotinib resistance is activated in the cell growth signaling pathway through heterodimer formation of MET and HER3 molecules. Thus, EGFR remains intact in *c-Met* amplification cells such as EBC-1, and erlotinib is able to inhibit EGFR phosphorylation. In the case of HCC827TR3, although the precise mechanism of resistance is not yet clear, it would seem that EGFR phosphorylation was inhibited by a similar mechanism. On the other hand, erlotinib could not inhibit EGFR phosphorylation in H1975 cells because the T790M mutation in EGFR causes a conformation change at the ATP binding pocket, thus decreasing the affinity between erlotinib and EGFR.

Since all of the erlotinib-resistant cell lines express EGFR, we examined the antitumor effect of combination therapy of docetaxel with erlotinib or irinotecan. In these models, erlotinib monotherapy did not show significant antitumor effect compared with the control group (Figs. 2A, 3 and 4). Interestingly, however, combination therapy of docetaxel with erlotinib showed a synergistic effect in HCC827TR3 (Fig. 2B) and EBC-1 (Fig. 3) xenografts. A similar result was obtained in HCC827TR3 xenografts using irinotecan as a chemotherapeutic agent (Fig. 2C). These results may indicate that the chemotherapeutic agent used in the combination therapy need not be restricted to a specific drug. On the other hand, no significant increase of antitumor effect of combination therapy compared with docetaxel monotherapy was observed in H1975 xenografts (Fig. 4). These results coincide well with the report of Okabe *et al* in which gefitinib and S-1 were used in combination in H1975 and HCC827GR5 xenografts (16). Since EGFR phosphorylation was completely inhibited by erlotinib in HCC827TR3 cells and EBC-1 cells but not in H1975 cells (Fig. 1C), it is possible that inhibition of EGFR phosphorylation is prerequisite for the combination therapy to be effective. EGFR phosphorylation activates signal transduction pathways, such as PI3K and Ras-ERK/MAPK, and erlotinib inhibits these pathways. However, the role of erlotinib in combination therapy in erlotinib-resistant xenograft models may be inhibition of signal pathway(s) other than the PI3K or Ras-ERK/MAPK pathways, because erlotinib monotherapy did not show any antitumor effect in HCC827TR3 and EBC-1 xenografts. In the H1975 xenograft model, erlotinib failed to inhibit EGFR phosphorylation (Fig. 1C) hence the antitumor effect of combination therapy was not enhanced. Okabe *et al* reported that the combination effect of S-1 with gefitinib was attributed to the down-regulation of thymidylate synthase (TS) by gefitinib and the mechanism could work even after the tumor cells became resistant to gefitinib (16). We consider that similar mechanisms are involved in our system, although the target molecules have so far not been specified. In the case of EBC-1, combination therapy using docetaxel was expected to reduce c-MET in cells, but this was not observed (data not shown). It was reported that erlotinib restores the effect of chemotherapeutic agents through direct inhibition of PgP or BCRP (17,18). However, this is unlikely because verapamil, a PgP or BCRP inhibitor, did not restore the sensitivity to docetaxel in HCC827TR3 cells (data not shown).

In our HPAC *in vivo* model which mimics PD in clinical therapy, the combination therapy of gemcitabine with erlotinib showed significantly strong antitumor effect compared with gemcitabine monotherapy (Fig. 5D). EGFR expression and phosphorylated EGFR were detected in the tumors of the control group after PD had developed. Surprisingly, phosphorylation of EGFR was completely inhibited in the tumors of the erlotinib group (Fig. 5C). These results indicate the usefulness of the combination therapy of a chemotherapeutic agent with erlotinib against *in vivo*-induced erlotinib-resistant tumors.

Erlotinib is currently approved for the treatment of NSCLC and pancreatic cancer. In the present study, we showed that combination therapy of a chemotherapeutic agent with erlotinib is efficacious against two erlotinib-resistant NSCLC cell lines (EBC-1, HCCC827TR3) and one pancreatic cancer cell line (HPAC) which had become erlotinib resistant, suggesting that this form of treatment would be useful against NSCLC and pancreatic cancer which developed PD. Erlotinib has been reported to have an excellent benefit for patients with NSCLC harboring mtEGFR and to prolong the overall survival of patients with NSCLC harboring wtEGFR (1,10). The combination therapy may be effective regardless of the EGFR mutation status because it was effective on both HCC827TR3 (mtEGFR) and HPAC (wtEGFR) cells.

The results suggest that combination therapy of a chemotherapeutic agent with erlotinib showed stronger antitumor effect compared with chemomonotherapy against erlotinib-resistant tumors in that erlotinib inhibited the phosphorylation of EGFR in the tumor. It may be possible to obtain evidence for the suitability of the combination therapy by monitoring the EGFR phosphorylation level in tumors after PD has developed following erlotinib treatment. However, this test cannot distinguish tumors which had intrinsically low EGFR phosphorylation and, to solve the problem, it may be necessary to test the EGFR phosphorylation level before the start of erlotinib therapy. In the present study, docetaxel, irinotecan and gemcitabine were used as chemotherapeutic agents. Whether or not similar results can be obtained with other chemotherapeutic agents is an issue for future research. If a patient goes into PD during combination therapy, a possible treatment modality may be to change the chemotherapeutic agent while continuing erlotinib.
